# Contracting for Catastrophe:Legitimizing Emergency Constitutions by Drawing on Social Contract Theory

**DOI:** 10.1007/s11158-021-09518-z

**Published:** 2021-06-16

**Authors:** Stefan Voigt

**Affiliations:** Institute of Law & Economics, University of Hamburg and CESifo, Johnsallee 35, 20148 Hamburg, Germany

**Keywords:** State of emergency, Martial law, State of siege, Emergency constitution, Constitutional political economy, Social contract theory

## Abstract

States of emergency are declared frequently in all parts of the world. Their declaration routinely implies a suspension of basic constitutional rights. In the last half century, it has become the norm for constitutions to contain an explicit ‘emergency constitution’, i.e., the constitutionally safeguarded rules of operation for a state of emergency. In this paper, I ask whether inclusion of an emergency constitution can be legitimized by drawing on social contract theory. I argue that there are important arguments, both against and in favor of constitutionalized emergency provisions, and that social contract theory—as applied by economists—can be of some help when deciding whether to have, or not to have an emergency constitution. This paper introduces a novel argument for justifying emergency constitutions. It argues that they can serve as a commitment mechanism protecting both citizens and politicians from overreacting to rare but significant threats.

## Motivation

At any point in time, a substantial part of the world’s population lives under a state of emergency declared by the government in charge. States of emergency frequently imply both procedural shortcuts (like government by executive decree rather than legislation passed by parliament), as well as substantive derogations of constitutional rights including basic human rights. Between 1985 and 2014, at least 137 countries declared a state of emergency at least once (Bjørnskov and Voigt 2018a). During the pandemic that hit the world in 2020, exactly half of all nation-state governments declared a state of emergency at more or less the same time (Bjørnskov and Voigt, 2020b).

It has not only become fashionable to declare states of emergency, but also to constitutionalize them. Today, some 90 percent of all countries have what I propose to call an ‘emergency constitution’. The way societies deal with emergencies has been (mis)used to argue that constitutional liberalism and the rule of law are not sustainable (most prominently by Carl Schmitt 1922).

Emergency constitutions are invoked when the stakes are very high. If their institutionalization is inadequate, catastrophe can result. It has been argued that the emergency constitutions prevalent in Latin America during the 19th century prepared the ground for the emergence of populist and military regimes in the 20th century because those constitutions allocated wide-ranging powers to the military that could easily be misused (Loveman 1993). Under emergencies, the number of innocent detainees and wrongly convicted people is often particularly high. Just think of the thousands of U.S. citizens of Japanese descent that were detained during WW II in the U.S.

In this paper, I ask two questions. First, can the passing of an emergency constitution be legitimized by drawing on social contract theory? Second, if it can, then what constitutional emergency rules would well-informed rational citizens consent to? To answer these questions, I draw on a social contract framework as commonly used by economists (most prominently by Buchanan 1975). I argue that arguments conventionally probed in favor of emergency constitutions are not entirely convincing and propose a novel justification for them. In times of unforeseen catastrophical events, both citizens and politicians tend to overreact, which may induce them to implement policies that are not suitable to face the respective challenge. In such situations, emergency constitutions may serve as a commitment device, protecting both citizens and politicians from their time-inconsistent preferences.

The remainder of the paper is organized as follows: second section first defines the term emergency constitution, and then describes central aspects of emergency constitutions in a bit more detail. Third section offers a short overview about the development of emergency constitutions over time, and also discusses their effects as identified by recent research in a brief summary of the current state of knowledge. Fourth section contains both a brief sketch and critique of the literature that justifies emergency clauses. Fifth section, the core of the paper, first discusses a number of reasons why rational individuals would hesitate to sign up to constitutionalized emergency provisions, and then advances a novel justification in their favor. Sixth section concludes.

## Emergency Constitutions: The Relevant Dimensions

A constitution has been defined as ‘a set of rules that is agreed upon in advance and within which subsequent action will be conducted’ (Buchanan and Tullock 1962, p. vii). This definition is very broad. Reducing the breadth of the definition to states and giving it an explicit economic dimension, one can describe constitutions as systems of rules used for the production of public goods that enable government representatives to make certain choices within previously fixed constraints that have been agreed upon in advance.

An emergency constitution can be described as a set of constitutional provisions that, under specific circumstances, empower governments to take action beyond the previously fixed constraints. At the same time, it limits governments’ use of extra powers, e.g., by defining core basic rights which can never be derogated or determining a maximum period of time over which government can resort to the additional powers. Note that emergency constitutions always contain a paradoxical element: a constitution defines the limits of state action whereas one of its parts—the emergency constitution—creates the foundations for suspending some of the limits created by the same document.

Historically, the primary rationale given by those who enacted emergency power provisions has been that those provisions would serve to ensure the survival of the state. This rationale refers to external threats such as war. Over time, emergency powers have been broadened considerably to be used during civil wars, revolutions, general strikes and subsequent to terrorist attacks.^1^ Often, a distinction is made between a French—subsequently Continental—approach to emergencies (*état de siège*), and a British approach originating from martial law (e.g. by Rossiter 2009, especially in Chapter X). Two aspects in particular make them distinct from one another. Namely, the identity of the actor authorized to monitor the state of emergency, and the degree of judicialization. Under a state of siege, monitoring would primarily fall to the legislature, whereas it would fall on the judiciary under martial law. Whereas the state of siege is legally anticipated, martial law could be characterized by ‘the absence of statutory foresight for its initiation and use’ (ibid., p. 141).

If the constitution is interpreted as ‘constituting’ the state, then the representatives of the state should be bound to the provisions of the constitution at all times, no matter whether normal or exceptional. This position can also be called monism. It implies the absence of any emergency rules. Under monism, citizens coordinate their behavior under a single set of rules. The rule of law applies to exceptional as well as to normal times, and basic human rights are not weakened during times of emergency. This view has been ascribed to both Leibniz and Condorcet, but also to Constant.

The alternative to monism is dualism, which comes in at least two types (Ferejohn and Pasquino 2004).^2^ According to the first, government behavior can and should be subjected to the constitutional order no matter whether the state of the world is ‘normal’ or ‘exceptional’. Under dualism, however, the relevant constitutional order to be applied depends on whether the state of the world is ‘normal’ or ‘exceptional’. Under this version of dualism, the competence of deciding on the concrete state of the world and, hence, on the *modus operandi* of the constitution is crucial, yet it does not lead ‘outside’ the rule of law and constitutional provisions.

**Fig. 1:**
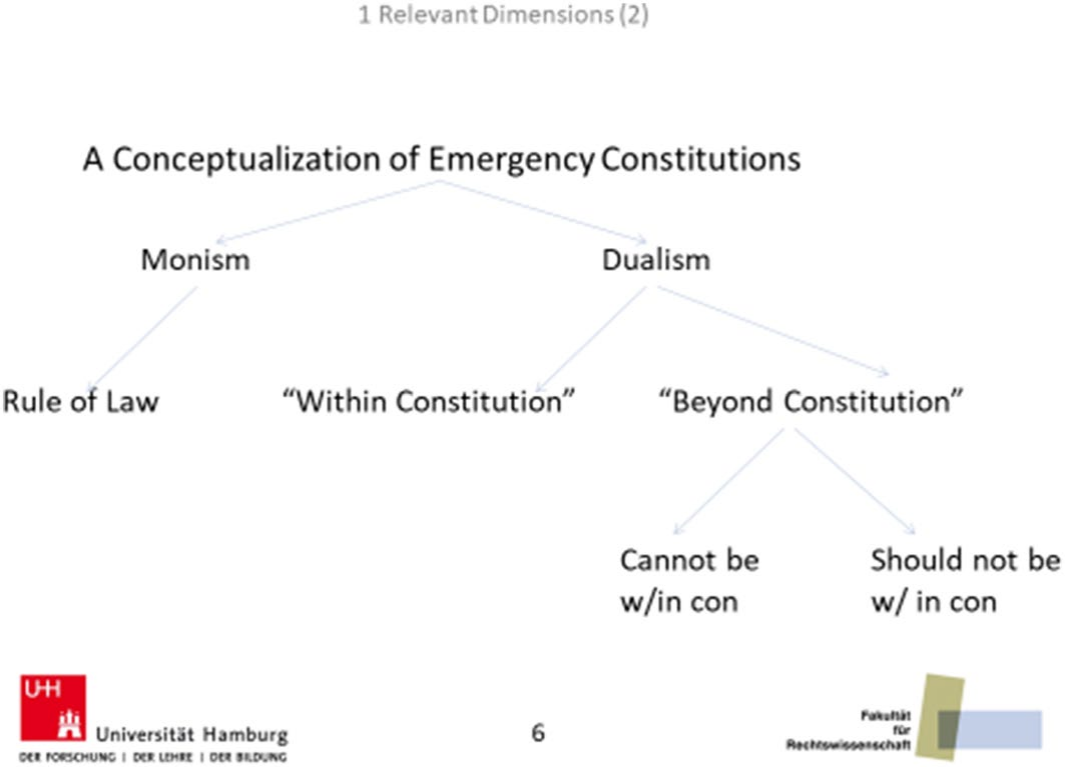
A conceptualization of emergency constitutions

The competence to decide on what state of the world applies is crucial also in the second type of dualism. Yet, adherents of the second version of dualism hold that states of emergency cannot be (or should not be) constitutionalized. Those who believe that it cannot be constitutionalized usually argue that human foresight is too limited to spell out the constraints under which government should operate under these conditions (most famously Schmitt 1922, p. 14). Those who believe that it should not be constitutionalized argue that this could be the best means to safeguard long-term constitutional fidelity (e.g., Gross 2003).^3^ This position also appears paradoxical: precisely because the rule of law is highly valued, it is proposed to be suspended temporarily. But this kind of dualism is silent on how to return to the desired state of affairs and the existence of situations in which politicians ought not to be bound by basic constitutional rules lets it appear rather incompatible with constitutionalism and the rule of law proper.^4^ At base, thinkers who share this position must assume that there are situations in which the rule of law is inept to sustain order in society. Figure 1 is a simple mapping of the approaches to emergency constitutions just discussed.^5^

Under monism, no rules to regulate a state of emergency (SOE), call them power-conferring rules, are needed.^6^This is also true of the second kind of dualism, at least regarding the kind and extent of competences that politicians have at their disposal. Since I am interested in the possibility of justifying an emergency constitution, only the first kind of dualism is of further interest here. Relevant institutional aspects that need to be dealt with under the first kind of dualism include:

(i) What are the necessary conditions for a state of emergency?
(ii) What (additional) competences does a state of emergency confer to the emergency government?
(iii) Who has the power to declare a state of emergency?
(iv) Who has the power to declare the end of an emergency?
(v) Who has the power to monitor the legality of the means used during a state of emergency?
(vi) Who exercises emergency powers?

In the following paragraphs, I briefly sketch the relevance of these aspects as well as potential institutional choices.

Ad (i): Emergency constitutions can be narrow or broad in the sense that they allow the declaration of a SOE only for a small or for a large number of events. If the constitution is precise in describing the events, this can lead to a low probability of a SOE being called.

Ad (ii): The probability of a SOE being declared is not only determined by the conditions named in the emergency constitution but also by the additional competences that it confers to a government under a state of emergency. One would expect constitutions conferring substantial additional powers to government to be attractive for government. Such constitutions might also be attractive for government if they can be easily misused: the right to dissolve parliament may, e.g., be misused simply to prolong one’s own stay in office.

Ad (iii): No matter how precisely the necessary conditions are defined, the power to decide must be allocated to some actors by power-conferring rules. At base, the question can be reduced to the degree of separation of powers in the declaration of states of emergency. On the one hand, one can imagine a constitution that allocates the power to declare an emergency exclusively to the head of the executive and no one needs to approve. This would be equivalent to a very low degree in the separation of powers. This is, e.g., the case in Kazakhstan. On the other hand, one can imagine that more than one constitutional actor must be involved in the declaration and more than one actor must approve it.^7^ In Liberia, e.g., the head of state declares but both chambers of parliament need to approve. An intermediate solution could be that some other branch needs to be consulted; if its advice is not followed, this might increase the political costs of declaring an emergency in terms of a reduction in perceived legitimacy.^8^

Ad (iv): Another important institutional issue is the power to end a state of emergency. One possibility (famously used by the Romans) is to have it expire automatically. Here, too, the separation of powers is central. Acknowledging that every state of emergency entails the danger of misuse with the possibility of developing into permanent autocracy, it seems to make sense to allocate the power to end a state of emergency to a constitutional actor other than the actor endowed with the exercise of emergency powers. Ackerman (2004) proposes a ‘supermajoritarian escalator’: the longer the state of emergency lasts, the more inclusive the parliamentary majority necessary to sustain it.

Ad (v): It seems natural to think of the judiciary as monitoring the legality of the means used. Yet, many scholars believe that speed is of the essence in emergency situations and that judicial review should be postponed (most prominently, of course, John Locke). Others, such as Ackerman (2004) or Dyzenhaus (2006), point to the dismal record of the judiciary in constraining government action under emergency.

Ad (vi): Conferring emergency powers to the head of the executive may be another natural assumption. Other provisions are, however, possible. They include not only the head of the military, but also technocrats. The French version of a state of emergency, the *état de siège*, implies an expansive delegation of powers to the military (Rossiter 2009, Chapter VI).

We now turn to different types of emergency constitutions as discussed in the literature. Ferejohn and Pasquino (2004) have proposed to distinguish between a ‘neo-Roman’ and a ‘legislative model’ of emergency powers that has close connections to the distinction between a monist and a dualist conception of legal orders. In Rome, government in normal times was led by two consuls. During states of emergency, which could be declared only by the Senate, the consuls would appoint a single dictator who could not be an active consul himself, but instead was recruited solely for the purpose of leading the emergency government for a maximum term of six months (Rossiter 2009, Chapter II). The dictator’s emergency powers differed from the powers of the consuls in that the dictator’s decisions were not subject to the veto powers of other magistrates nor to the intercession of the tribunes.^9^ The Roman institutions were, hence, built on a dualist conception. Some modern constitutions do vest an elected president with extraordinary powers. The main difference to ancient Rome, of course, being that the dictator had to be a person who was not part of government during normal times. As examples of the neo-Roman model, Ferejohn and Pasquino (2004) name the Weimar Republic and the current French Constitution.

The ‘legislative model’, by contrast, is characterized by the legislature passing statutes to fight the emergency. It is, however, built on a monist notion of the legal order. Ferejohn and Pasquino (2004, pp. 215–217) cite the various Defence Acts Against Terrorism passed by the British parliament and the PATRIOT Act in the U.S., but also the various laws passed in the 1970s and 1980s in both Italy and Germany to deal with terrorism. The dualist approach implies the vesting of extraordinary powers that will be divested after the state of emergency has ended. It also implies that any legislation produced during the emergency for the purpose of fighting the emergency, will be invalid after the emergency has ended. This is different if an emergency is fought by passing ordinary legislation.^10^ In other words, monism entails the danger that civil liberties will be derogated not merely for a fixed period of time but will be suspended for good. It would, of course, be interesting to compare the effects of the neo-Roman with those of the legislative model, but this must be left to another paper since here, I am interested only in the possibility of justifying an emergency constitution.

Our inquiry into the possibility of establishing justification for an emergency constitution is based on two aspects. The first aspect asks whether an emergency constitution is the most suitable means to reach a certain end. It draws on available social science in making this judgment. The second one asks whether an emergency constitution can be justified in the sense of being recognized as legitimate by all those living under an emergency constitution. The first of these aspects is dealt with in the next section of the paper whereas the second aspect occupies center stage in Sect. 4 and 5.

## Emergency Constitutions: What Effects do they have?

When discussing how the constitutionalization of emergency provisions could be legitimized, we assume that this is done taking into consideration the current state of knowledge regarding the effects of emergency constitutions. Before offering a brief summary of the current state of knowledge, some basic information on the development and prevalence of emergency constitutions might be helpful (Bjørnskov and Voigt 2018a, pp. 103–106 provide a more extended overview).

**Fig. 2:**
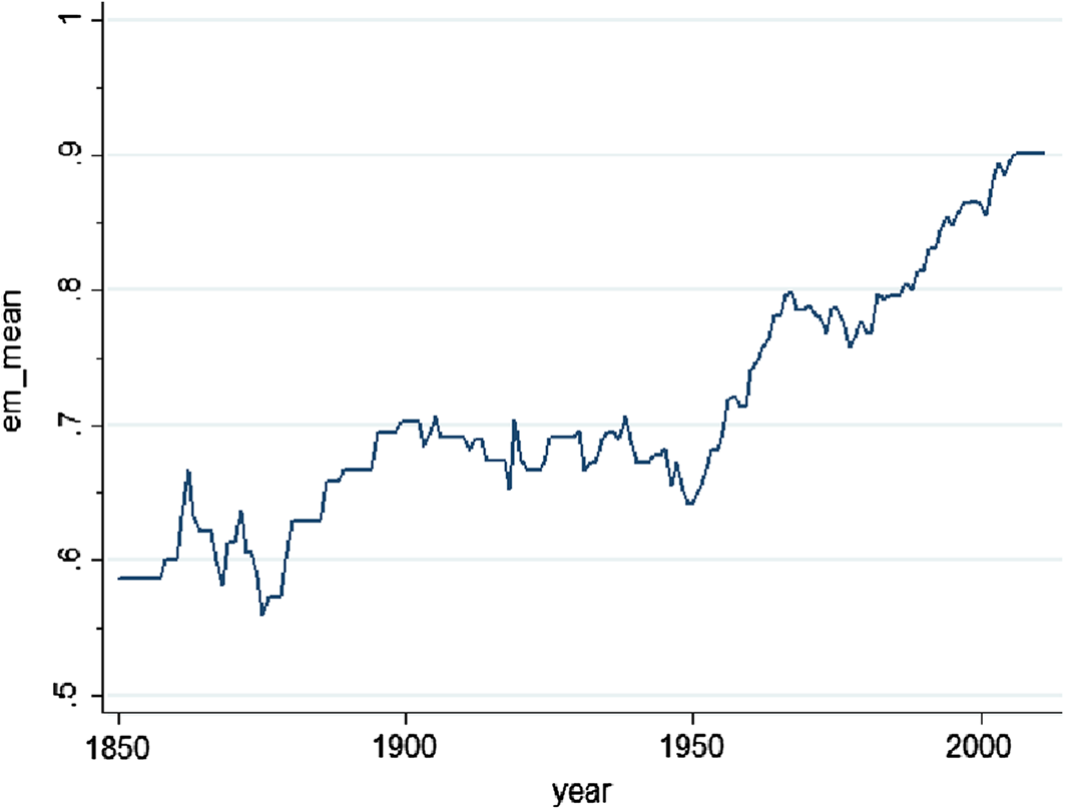
The proportion of countries having an emergency constitution. *Source* Comparative Constitutions Project. Exact wording of the variable: ‘Does the constitution have provisions for calling a state of emergency?’

At the end of World War II, around 70 percent of all countries had an explicit emergency constitution.^11^ The proportion increased to around 90 percent by 2000 and has remained there ever since, as shown in Fig. 2. Moreover, ever more conditions are included in constitutions to justify the use of emergency powers. In other words, it has become easier to call a state of emergency.^12^

In a number of recent papers, Bjørnskov and Voigt discuss the effects of emergency constitutions. Bjørnskov and Voigt (2018a, pp. 111–115) introduce an index of emergency powers (INEP) that measures how difficult it is to declare a state of emergency, as well as the benefits a government enjoys once it has declared a state of emergency. Both the cost and benefit side of the INEP include three variables.The three variables depicting the difficulty of calling a state of emergency and, hence, the cost side, are how many actors are needed to declare a state of emergency, how many actors need to consent to the declaration for it to become valid and how many different situations are explicitly mentioned in the constitution that warrant the declaration of a state of emergency. To construct the benefit side, it is asked whether fundamental civil and political rights can be suspended during a state of emergency, whether parliament can be dissolved and whether government can introduce censorship of the media or expropriate property during an emergency. Using a scale between 0 and 1, Bjørnskov and Voigt (ibid.) show that constitutionalized emergency powers have risen from an average of .23 in 1950 to almost .35 in 2011, thus documenting a significant increase in powers allocated to the executive over these six decades.

The INEP is used to ascertain whether differences in constitutional emergency provisions have any consequences. It turns out that they do. In a paper dealing with the determinants of declaring a state of emergency, Bjørnskov and Voigt (2018b) find that the easier it is to declare a state of emergency, the more such declarations occur. They also ask whether not having an emergency constitution at all has an impact on the likelihood that an emergency will be declared. They find that countries without constitutionalized emergency provisions are significantly more likely to declare a state of emergency, ceteris paribus.

Inquiring into the effects of natural disasters, Bjørnskov and Voigt (2018c) find two disturbing results. First, the more benefits the executive enjoys as a consequence of declaring a state of emergency, the more people die subsequent to a natural disaster (the severity of the disaster is controlled for by taking the number of people affected explicitly into account). The second finding is that the less costly it is to declare a state of emergency, the larger the negative effects in terms of deteriorations in the quality of basic human rights.

Natural disasters are largely exogenous events. The effects of declaring a state of emergency subsequent to such disasters might be considerably different from the effects of emergency declarations following terrorist incidents. Terrorist activities are usually aimed against the government in place. In that sense, they are endogenous because their likelihood depends on the government in place and the policies it pursues. They are also endogenous because they might already be responses to counterterrorist policies enacted by government. Bjørnskov and Voigt (2020b) find that states of emergencies declared as a response to terrorist attacks are accompanied by increased government repression. It is, however, entirely unclear whether increased government repression leads to fewer terrorist incidents. Preliminary results seem to indicate the exact opposite. This may be a consequence of increased alienation of relevant groups induced by repressive measures. To prevent terrorist plots from reaching fruition, the police and other security agencies depend on information revealed to them by friends and familiy of potential terrorists. But if these informants are alienated as a consequence of increased government repression, they may no longer be willing to provide the police with the necessary information.^13^

In sum, emergency constitutions clearly influence politicians’ behavior. The easier it is to declare a state of emergency the more such declarations occur. The easier it is to declare, the more basic human rights are likely to suffer from a declaration. This sheds serious doubts on the suitability of emergency constitutions to deal with large unforeseen events. In turn, justifying them becomes a true challenge.

## Justifying Emergency Legislation: A Brief Sketch of the Contractarian Literature

Much recent discussion has defended or even praised Locke’s prerogative which can be interpreted as a proposal to deal with emergency situations in an extra-legal way (Lazar 2009 but also Pasquino 1998, and Casson 2008). This is why I briefly summarize and critically discuss Locke’s (1824) approach in this part of the paper.^14^

Before discussing Locke’s argument, it makes sense to look at Machiavelli’s thoughts because it was he who ‘rediscovered’ the institutions used in ancient Rome for emergencies (although Machiavelli was, of course, not a contractarian). In the *Discourses on Livy*, Machiavelli writes: ‘… those Republics which in urgent perils do not have resort either to a Dictatorship or a similar authority, will always be ruined in grave incidents’ (*Discourses*, Book 1, Chapter XXXIV). Machiavelli reveals himself as a great admirer of the Roman institutions. The Roman model plays a central role in the justification of emergency constitutions to this very day (see, e.g., Rossiter 2009 or Ferejohn and Pasquino 2004). Ever since Machiavelli, two arguments have been frequently used to justify them: the lack of time to respond to an emergency using the established procedures and the incompleteness of ‘the laws’ due to restrictions of the human capacity to anticipate the future.

Regarding the first argument, many philosophers and political theorists argue that speed is of the essence in emergencies and that no time must be wasted in such situations. Since collective decision-making requires more time than individual decision-making, in order to speed up the decision-making process, one individual should make all the important decisions. This argument can, for example, be found in Machiavelli (1531) who presents it as a two-step argument: (i) collective bodies take more time than individual persons to decide, and (ii) the separation of powers also slows things down. Interestingly, the costs of the simplified decision-making endorsed via emergency constitutions (e.g., in terms of bad decisions) are seldom discussed although Machiavelli is clearly aware of the potential costs, warning that relying on ‘the extraordinary method’ could serve as pretext for evil objectives (ibid.). Harrington (1992), who writes about the dangers of ‘unnatural haste’, is certainly the exception.

Regarding the second argument, Locke maintains that the executive is in need of discretion as legislators are unable to foresee every possible contingency (*Two Treatises on Government*, II, §160). With this, we have hit Locke’s famous prerogative which is to apply to circumstances not foreseen by the legislators including states of emergency (Locke does not use this term but speaks of ‘accidents and necessities’ [ibid.]). In §160 of his *Second Treatise*, Locke describes prerogative as the ‘power to act according to discretion, for the public good, without the prescription of the law, and sometimes against it’. Casson (2008, p. 954) summarizes Locke’s point as follows: ‘Prerogative is a power that exists beyond written law, yet it is constrained by constitutional authorization. It is in this sense that Pasquino (1998, 19) can assert that Locke provides us with “a systematic constitutional theory of extralegal power’’’. This position is difficult to reconcile with Locke’s statement quoted above: ‘without the prescription of the law, and sometimes against it’.

John Locke is hailed as a proponent of constitutionalism and the rule of law. This is why his reliance on the prerogative has been criticized by many as it seems incompatible with the rule of law. Fatovic (2009, Chapter two) tries to reduce the seeming incompatibility by reminding us of the paramount importance that Locke attributes to natural law. According to Locke, the laws of nature are ‘permanent, unvarying, inerrant, and inviolable’ whereas human laws are ‘historically contingent, changing, fallible, and potentially breakable’ (ibid., p. 61). The overarching goal of natural law is ‘the preservation of society, and (as far as will consist with the public good) every person in it’ (II, § 134). It is natural law that is to constrain the executive in its use of the prerogative. Since natural law and civil law can deviate from each other, it is the function of the executive to ensure that natural law will be complied with, if needs be by relying on the prerogative. This position takes the perceptability of the natural law for granted.

Locke conceives of two mechanisms to constrain the executive in its use of the prerogative, namely (1) virtue and (2) the right to resistance by the governed. His view of virtue is instrumental in the sense that he thinks princes to be virtuous because they are afraid of being punished, e.g., by being removed from power. Locke is, however, aware that the right to resistance may not be perfectly reliable to prevent tyrannical misuse of the prerogative, writing that many misuses ‘will be borne by the people without mutiny or murmur’ (II, §225).

To this day, Locke is often mentioned as having developed a convincing justification for emergency provisions. Casson’s (2008) explicit defense of Locke at the expense of Schmitt is only one example of this. My own reading of Locke is much more critical. From a 21st-century perspective, informed by both empirical evidence on how emergency powers have been used and a public choice perspective modelling governments as maximizing their own utility, the Lockean treatment of the ‘public good’ is not convincing. True, the pursuit of the public good is mentioned as a condition for the legitimacy of prerogative powers, but who is to be the judge on government behavior? Locke even wants to make the scope of prerogative powers a function of the character of the individual wielding it (Fatovic 2009, p. 41). Who is to judge government’s character? As just cited in the last paragraph, Locke himself was not entirely convinced of the reliability of the right to resistance as a mechanism to keep government in check.^15^

Social contract theory received another boost exactly 70 years later. Rousseau (2019) is an excellent representation of some of the arguments regarding states of emergency that are still quoted today. His two main arguments are speed and the incompleteness of legislation. In *The Social Contract*, he deals with the latter issue writing: ‘A thousand cases can arise for which the Lawgiver did not provide, and it is a very necessary foresight to sense that one cannot foresee everything’ (IV, 6, 1).

But he still held that only the greatest danger, namely when the existence of the country is at stake, could justify the temporary non-application of the laws. He was very optimistic regarding the recognition of such situations (‘in these rare and obvious cases’). He sketched two models of government under emergency, depending on the ‘nature of the danger’. The first and more moderate model would not entail a change in the laws, only in the way they are administered. The second model, on the other hand, suggests nominating a supreme ruler ‘who shall silence all the laws and suspend for a moment the sovereign authority’. In such cases, there would be ‘no doubt about the general will’. He traces both models back to Rome, but insists that Rome borrowed the second model from Alba. To prevent the misuse of emergency powers, Rousseau proposed to impose a number of limitations on them. One such limitation was that the dictator was not allowed to legislate (IV, 6, 4). He also insists that the time period allocated to a dictator be ‘a very brief term that can never be extended’ (IV, 6, 11).

Today, Rouseaus’s assertions seem optimistic and even naïve. They are optimistic because he seems convinced that the evaluation of the concrete situation will be more or less unanimous (stating that there is ‘no doubt about the general will’), and they are naïve because he wants the ‘most worthy’ to be entrusted with the special powers. Locke, unfortunately, remains completely silent on procedure, i.e., rules according to which a normal state can be transferred into an exceptional one, and then returned back to a normal state. With hindsight, Rousseau’s assertions also appear optimistic and naïve because it has been shown that emergency provisions have not only been misused frequently (Bjørnskov and Voigt 2021) but have served as a welcome instrument of would-be autocrats to put their countries on a path away from the rule of law and democracy (Lührmann and Rooney 2020).^16^

Of course, there are philosophers who were convinced that societies are able to frame rules of such generality that no emergency constitution is necessary. These include Tocqueville and Constant. Instead of summarizing their arguments here, I would like to mention Scheppele’s (2004) point that the standards for legitimizing states of emergency should be higher under republican forms of government than under monarchies. Many of the justifications summarized here were written with some sort of prince in mind. In a modern constitutional democracy, some of these arguments therefore no longer hold.

## A Novel Justification of Emergency Constitutions

A social contract argument used to justify both the existence of the state and to legitimize specific constitutional rules has been popularized among economists by James M. Buchanan (1975). According to him, the purpose of the contractarian approach is justificatory in the sense that

‘it offers a basis for normative evaluation. Could the observed rules that constrain the activity of ordinary politics have emerged from agreement in constitutional contract? To the extent that this question can be affirmatively answered, we have established a legitimating linkage between the individual and the state’. (1987, p. 249)

In this section, I ask whether the observed emergency constitutions could have emerged from agreement in constitutional contract. This section is divided into four parts. I begin by rehearsing the economic perspective on the social contract view. I then set out the basic assumptions used for the justificatory exercise. The third part presents a number of reasons why rational individuals are unlikely to consent to an emergency constitution. The fourth part revisits this notion of consent, and finds some reasons why rational individuals might very well agree to constitutionalized emergency provisions.

### The Economic Take on the Social Contract

In their *Calculus of Consent*, Buchanan and Tullock (1962) note that consent is scarce and that this fact would justify an economic analysis of constitutions. In the *Limits of Liberty*, Buchanan (1975) offers a rationale for leaving the state of nature or anarchy behind and moving on to a constitutional state. This move would shift realized utility levels of all those living in that state outward and closer to the utility frontier. The utility that can be realized in the state of nature has the important function of serving as a fallback option. No rational individual would ever accept a constitution under which her (expected) utility would be lower than under the state of nature.

Here we are primarily interested in the question of whether unanimous consent to a particular part of the constitution—the ‘emergency constitution'—appears plausible. This does not mean that questions of implementation should be set aside lightheartedly. This is why I ask to what degree the emergency constitution—if it can be legitimized at all—can be expected to be self-enforcing. Self-enforceability would be high if political actors have incentives to control each other.

The ‘original social contract’ is a disarmament contract that endows the representatives of the state with immense power that could easily be misused. The institutional set-up requires that the probability of misuse is somehow contained. The separation of powers, both horizontally and vertically, is the most important such institutional device. Holding elections at regular intervals could be another one. But democracy also entails numerous dangers such as the tyranny of the majority. One means to contain those dangers are basic human rights.

In this context, basic human rights can be given a very simple rationale. Negative rights can be interpreted as creating protected domains that not even the state is allowed to trespass, and can be thought of as part of the original contract. In short, individuals are only willing to disarm themselves on the understanding that they are endowed with protected domains into which the state will not enter. For democratically organized states, this implies that negative rights limit the possible scope of majority decision-making. If rights are really basic, even large majorities are bound by them and cannot simply ignore them, nor infringe upon them. Negative rights can thus also be interpreted as a device to protect minorities against current majorities, or as ‘veto rights'. In this sense, they give the holders of these rights the right to behave in a certain way, even if an overwhelming majority would prefer them to not to act in that way.

One argument for justifying basic human rights could be that individuals who choose a basic legal framework from behind a veil of uncertainty are uncertain about their own individual position in the future. They might belong to a minority and might not be willing to submit to the will of the majority. If enough individuals consider this probability to be sufficiently large, they have an incentive to create basic human rights. Human rights can thus be interpreted as insurance against adverse effects on one’s own utility given that one finds oneself to be with the minority. This is, of course, only one of many possible justifications for basic human rights. But it is based on economic reasoning: being forced not to behave according to one’s preferences is utility-reducing and costly. Agreement to basic human rights can thus be the result of a calculus based on exchange.

To sum up, it has been shown that the social contract approach can be given an economic logic.^17^ This was shown in particular with regards to negative basic rights. In all likelihood, an emergency constitution would enable a government to derogate from some of these basic rights. Whether rational individuals are likely to agree to such derogation is, of course, the central question that will be taken up in the third part of this section.

### Assumptions

Here, I am primarily concerned with the amount of information that the individuals choosing—or not choosing—their emergency constitution have. The Rawlsian veil of ignorance has been criticized for assuming that the choosing individuals ‘know everything in general but nothing in particular’ (Cooter as cited in Buchanan 1977, p. 205) in the sense that they are perfectly informed about cause-effect relationships of many institutions, but have no clue regarding their own selves. The question thus is: How much do we know ‘in general’, or rather, how much knowledge do we assume that those who are choosing their basic rule-set have at their disposal?

Speaking of the ‘veil of uncertainty’, Buchanan and Tullock (1962, p. 78f.) assume that people know who they are today but they are uncertain about the future and their future selves. Whatever merits the Rawlsian veil of ignorance approach may have, I want to side with Buchanan and Tullock and assume that individuals are familiar with emergency measures taken as a reaction to prior emergencies. We know the consequences of Art. 48 of the Weimar Constitution, we know how U.S. citizens of Japanese descent were treated during World War II, we know that many suspects have been detained in Guantanamo as a consequence of 9/11. Moreover, we know the current state of technology, and we also know the costs of the measures enacted as a consequence of, e.g., 9/11. Finally, we assume that people also know whether their country is disaster-prone. Formulated differently, I want to assume that the insights regarding the effects of emergency constitutions as summarized in Section 3 above are known to all of the decision-makers behind the veil. All the information the decision-makers are assumed to have is based on previous events; they are, hence, not endowed with any clairvoyant capacities.

Further, I assume that the entire rest of the constitution has already been agreed upon. On the one hand, this assumption is a deviation from the approach chosen by Buchanan where the constitution is chosen in its entirety. On the other hand, it simplifies the analysis as the number of decisions to be taken simultaneously is dramatically reduced.

### Trying to Justify an Emergency Constitution

Given the assumptions just discussed, would rational individuals opt in favor of an emergency constitution? As we have seen in the last section of this paper, the two most frequently offered justifications for an emergency constitution are the need for speed and the impossibility to cover all future contingencies under general constitutional rules.

The argument that the life of a nation could be threatened because its constitutional organs are not fast enough in making decisions comes in two varieties. The first one claims that collective decision-making is much slower than individual decision-making. The second argument holds that in times of crises, making decisions could be impossible because parliament is not in session, legislators could not meet, etc.

Let us address them in turn. The first variant does not seem to be convincing: If the life of the nation is threatened, both the legislature as well as the executive are likely to act more cooperatively than in non-emergency times and to agree on some measures immediately. Just remember the passing of counter-terrorist legislation after 9/11. The U.S. PATRIOT Act which is more than 300 pages long was passed a mere 45 days after the events. Other countries were almost as quick: the British parliament passed the Anti-terrorism, Crime and Security Act on November 19. The Canadian Anti-terrorism Act became law on December 18 after having received Royal assent.^18^ But if the survival of the nation is at stake, unusually high levels of cooperation are not confined to the legislature. During World War I, Britain had a coalition Cabinet—at base completely alien to a majoritarian electoral system—which was created to enhance government’s legitimacy. A War Cabinet was created to speed up decision-making (described in Rossiter 2009, Chapter XI). Yet, executive acts that need not be authorized by the legislature—such as certain executive decrees—still require less time than any kind of cooperation across government branches.

The second argument claims that in times of crises decision-making could simply be impossible, e.g., because parliament is not in session. Whereas the first argument cannot be dismissed lightly, this one is not convincing in the 21st century anymore. In monarchies in particular, parliament was often only in session at the behest of the monarch. During the 19th century it was not uncommon for a country’s parliament to be in session only for two or three months every year. But this is no longer the case. During the pandemic of 2020, many parliaments created the option to assemble only virtually, i.e., online.

The argument that constitutional rules being passed today could be insufficient or inappropriate for tomorrow’s emergencies has been made by Schmitt (1922, p. 6f.).^19^ Today, terrorism is often described as a qualitatively new phenomenon that old constitutions would be ill-equipped to deal with. This argument is unconvincing for two reasons. First, terrorism has been around at least since Spanish guerilla fighters were fighting Napoleon’s army in the early 19th century. Second, the average life-span of constitutions is less than 20 years (Elkins et al. 2009, pp. 129–146). In other words, the phenomenon of terrorism is much older than almost all constitutions in place today.^20^ Flipped around: on average, each society has had the chance to modernize its emergency provisions every 20 years over the course of more than two centuries. The probability of constitutions being completely outdated and out of sink with contemporaneous threats appears, hence, rather low.

One important trait of constitutional rules is their generality, which makes them applicable to a wide variety of situations that could not possibly have been foreseen at the time they were passed. This can even be interpreted as a pragmatic rationale for making rules as general as possible. Granted, our imagination may fall short of imagining the concrete issues at stake for extreme and highly improbable contingencies—and the COVID 19 pandemic comes to mind immediately—but does this justify special rules that are only applicable during times of emergency?

In sum, it is unclear how to justify a dualistic approach to the constitution that differentiates between normal and extraordinary times. Yet many constitutions do. And international law does, too. So, let us analyze the rationale offered in favor of the possibility to derogate from a number of basic human rights, such as freedom of assembly and association, freedom of movement, freedom of speech and electoral self-determination. Unfortunately, such a rationale is rarely made explicit. At base, derogation clauses seem to imply that respect for human rights is a sort of luxury that is too costly to afford in times of crises.^21^ This position is, of course, at odds with the argument here developed: basic human rights are not a luxury good but a precondition for unanimous consent to the disarmament contract. One reason for calling them basic is precisely their non-derogability.^22^

Among economists, such a complete refusal of any tradeoffs regarding basic human rights might not be the norm. An alternative take that is often used by legal scholars would ask whether derogations from human rights are at all proportionate. But to prevent us from falling back into a more conventional welfare-based type of analysis, the question is not answered with reference to any traditional welfare standard but by asking whether a hypothetical member of the constitutional assembly would be willing to forgo the benefits of a strict protection of constitutional rights in favor of some possible benefits regarding a particular emergency situation. If one can imagine that all members of a constitutional assembly—or even all members of society—are ready to forgo some constitutional rights protection in favor of some benefits regarding an emergency, we would have established a legitimating link between individual citizens and an emergency constitution.

But before the issue of proportionality can be meaningfully addressed, we must pose another question. Namely, is the suspension of any right at all suitable for successfully dealing with the emergency?

During World War II, the U.S. detained many citizens of Japanese descent, predominantly on the West Coast. The Supreme Court held this practice to be constitutional.^23^ Their detention was equivalent to the suspension of many aspects of due process or habeas corpus. In hindsight, it seems more than questionable whether this disrespect for human rights improved the odds of the U.S. winning the war, of winning it faster or causing fewer casualties. In that sense, the measure was unsuitable. With this knowledge available today, it is questionable whether rational individuals would be in favor of allowing the possibility of softening due process during times of emergency.^24^

This is a single example. But the suitability question can be raised on a more general level: terrorist attacks are most likely to be prevented if friends and family of would-be terrorists turn to the police to warn of planned attacks. Walsh and Piazza (2010) argue that this is less likely if the police are known not to comply with physical integrity rights. Bjørnskov and Voigt (2020b) find that increases in repression levels subsequent to a terrorist attack lead to more, rather than less, terrorist incidents, hence presenting evidence that some measures made possible by emergency constitutions are unsuitable to fight terrorism. But suitability is not a sufficient criterion, it is merely a necessary one. Given that a measure is suitable, it also needs to be proportionate.

To illustrate the case of proportionality let us assume, hypothetically, that detention of 800 Muslims would lead to the identification of one active member of the so-called Islamic State and the detention was somewhat suitable. But the measure would imply that 799 innocent people were detained. This case is, of course, not only hypothetical as up to almost 800 Muslims were held by the U.S. in its Guantanamo detention camp. Would this be proportionate? To frame the question in terms of a constitutional assembly: Would I agree to live in a country that can detain a very high number of innocent people? Suppose I were a member of a minority group. Would I consent to the possibility of being detained for an indefinite period knowing how people of Japanese descent were treated before? Given that the answer is no, no consent for the derogation of human rights in times of emergency can be assumed.^25^, ^26^

Summing up, many considerations seem to speak against emergency provisions that allow the government to derogate from basic human rights. Moreover, the time pressure argument frequently offered in favor of shifting powers away from the legislature (and the judiciary) to the executive is not convincing in the 21st century. But the shift in competences and the possibility of government to intrude into the private sphere of its citizens are the two core elements of emergency constitutions. Viewed like this, attempting to legitimize them on the basis of social contract theory seems futile.

### Another Attempt to Justify Emergency Constitutions

An attack like 9/11 can make many people very angry. Just having experienced an extreme condition can lead them to demand drastic measures. Once calmed again, it might turn out that these measures were extremely costly, disproportionate or even outright unsuitable. If, subsequent to a terrorist attack, voters demand tougher counter-terrorism policies, governments are likely to supply them because they want to be re-elected. These policies might, however, be unsuitable as they can induce additional alienation between government and communities from which terrorists might emerge. If entire communities get alienated, their members are unlikely to report suspicious activities which could actually make terrorist incidents more, rather than less, likely.

Rational citizens might, hence, want to protect themselves against succumbing to the danger of ‘action bias’ too easily.^27^ Action bias has been described as a consequence of probability neglect: since many individuals focus on the bad outcome of an event and not on its likelihood of ever occurring again, they overreact. Action bias can be observed in individual behavior (not taking the subway to the airport any more after an attack on a subway) but also in demands that government show a tough response. Since action bias is a very prominent reaction, governments have incentives to react strongly to keep own popularity levels high.

We have thus arrived at Ulysses and the Sirens, one of the most basic allegories in constitutional political economy. In more modern language, people know that they are subject to problems of time inconsistency. In ‘normal times’, they know that a carefully crafted reaction to any problem promises the highest return. Yet, confronted with an emergency, they tend to overreact and fall prey to action bias which could also be called the ‘we need to do something now fallacy’. If, in a calm mood, they anticipate this possibility and realize its potentially huge costs, they may want to take care to implement precautions against it in the constitution. Here, the constitution could serve as a commitment device that prevents politicians from giving in to the tempting voices of the Sirens (in this case their potential voters). Possible ways to institutionalize such precautions include the requirement that legislation can only be passed after so many readings in parliament, that a minimum amount of time must pass between readings and so on.

So, if constitutions are seen as a commitment device, and members of society believe they are subject to the ‘we need to do something now fallacy’, then this constitutes a strong argument in favor of including emergency provisions in a country’s constitution. Notice that its function is entirely different from the one perceived by Ackerman (2004). For him, it served primarily as an enabling device (reassuring that government was acting) whereas here, it serves to constrain government (‘do not do anything you might regret tomorrow’). If this argument and the evidence presented above are considered jointly, a number of implications follow:

- The hurdles for invoking a SOE should be high, implying that various actors need to agree. Only if there is broad consensus across factions that the country is challenged by an emergency should it be possible to switch from the ‘normal’ to the ‘emergency state’. The high hurdles are to protect the country against governments that try to misuse the emergency provisions to their own advantage.
- The attempt to exactly specify the conditions under which a SOE can be invoked is of little use when uncertainty is genuine. Whether cyber war attacks are sufficiently serious to be included in the constitution is hard to predict. Rather than specifying the conditions, increasing the number of actors who need to agree seems more sensible. The actors that need to consent would depend on the specific constitutional tradition. Beyond the chambers of parliament, they could include a court, expert bodies, but possibly also the citizens if the country has a strong tradition in direct democracy (and time allows).
- The additional benefits a government enjoys after declaring a SOE should be strictly limited. It is unclear why dissolving a parliament could ever help to resolve an emergency more effectively.
- This also holds for the possibility to derogate from basic human rights. There is evidence that many such derogations are unsuitable and, hence, ineffective.^28^
- Measures passed under a SOE should automatically cease to be valid once the SOE is over.
- All decisions connected to the declaration of a SOE and the policy decisions taken under it should be subject to judicial review.

Since there is evidence that emergency constitutions do affect government behavior, spelling out the constraints as precisely as possible does promise to have the intended effects. Involving all branches of government at some point also increases the chances that the emergency provisions will be self-enforcing since representatives of each branch can be expected to guard their own interest against intrusions from either of the other branches.

Constitutionalizing emergency provisions implies a clear separation between normal and emergency times. Under a monist legal order, newly created legislation often survives the emergency proper and becomes part of the permanent legal order. Measures reducing civil rights can therefore lead to a perpetual decline in the level of basic human rights. If measures passed under a SOE are only valid until the end of the SOE, a dualist order of the first kind might be better able to safeguard civil rights over time. In addition to the institutional safeguards just spelled out, rational individuals are therefore likely to demand that measures passed under a SOE become automatically void once the emergency is over.

Human foresight is limited. Catastrophic events not foreseen by anybody may require swift government action. But acknowledging the human tendency to overreact would make rational individuals favor an emergency constitution that does not only enable governments to react swiftly but tries to minimize the possibilities of misuse at the same time. Once it has been shown that a dualist approach can be justified, the question becomes: What kind of emergency constitution? I hope I have shown that the involvement of a high number of veto players is desirable, and not just because it increases the chances of the self-enforceability of an emergency constitution. While the consent of many players for declaring a state of emergency seems desirable, relatively few benefits should be offered to the government under a SOE. And finally, the *ex post* accountability of government should make forward-looking governments more careful in the choice of policy options.

## Outlook: Toward an Economics of Emergency Constitutions

This paper has been confined to a single question, whether emergency constitutions can be justified from a strictly individualistic perspective. The question has been answered in the affirmative, but with important caveats.

The question dealt with in this paper is a normative one. The fact that constitutional provisions that put few constraints on government have become the norm over the course of the last five or six decades is a positive observation. The divergence between our justificatory exercise and political reality is easy to explain. For many emergency constitutions, (hypothetical) consent is hard to imagine. Instead, their creation is likely to have been in the interest of the ruling elites.^29^ Emergency constitutions can be very valuable for ruling elites even if they are never actually used; the mere hint at the possibility of using them might already be sufficient reason for the opposition to moderate its behavior.

Some follow-up questions come immediately to mind:

1. Is the presumption that constrained dualism strictly limits the application of laws passed during SOEs to emergency periods empirically correct? Are there systematic differences in both the kind as well as the quantity of anti-terror legislation passed by countries following either of the two main models described in this paper?
2. Implicitly, we have assumed throughout that the decision to incorporate emergency provisions into a constitution takes place in an environment where the entire remaining text of the constitution follows well-established rule-of-law principles, and has a high chance of being implemented according to the letter of the law. But, assume we are dealing with a newly independent state or a fledgling new regime. In that case, it might very well be possible that emergency constitutions are misused by governments in order to stabilize their position. It might, hence, be necessary to add an important qualifier to the normative argument here developed.

## Footnotes

1 Interestingly, this ‘broadening over time’ seems to repeat itself as it took already place in Rome. A number of researchers (Lintott, 1999, p. 111; Straumann 2016, p. 72f.) argue that, at least originally, the Roman dictatorship was confined to external threats and could not be applied to fight domestic unrest. A similar broadening occurred again in the 20th century and is described in more detail in Bjørnskov and Voigt (2018a).

2 They also distinguish between monist and dualist conceptions but would group my first dualist conception into the monist camp.

3 Gross proposes to call this the ‘extra-legal measures model’. It would inform ‘public officials that they may act extralegally when they believe that such action is necessary for protecting the nation and the public in face of calamity, provided that they openly and publicly acknowledge the nature of their actions’. Apparently, no (constitutionalized) actor is charged with monitoring if public officials have ‘openly and publicly’ acknowledged the nature of their actions. In other words, the status of this provision remains unclear.

4 An advantage of hardcore dualism could be that it is clear from the outset that the emergency government cannot produce any legislation that will be valid beyond the state of emergency.

5 This is, of course, only one of many possible mappings. Loevy (2016) proposes to distinguish between the extralegal Lockean model, the neo-Roman model of dictatorship and the rule-of-law model of legality.

6 This does not exclude the possibility that specific competences are allocated to government on the basis of statutory law. It was mentioned in the introduction that today, some 90 percent of all countries dispose of an emergency constitution as defined here. The remaining ten percent have passed statutory legislation to deal with emergencies. In fact, many—if not most—countries having emergency constitutions also rely on statutory law in addition to their constitutional provisions. An example is public health laws on whose basis many governments imposed quarantine on their populations during the COVID pandemic, often without declaring a state of emergency. Counter-terrorist legislation is another area in which much statutory law exists.

7 Throughout the paper, I will refer to this as separation of powers. Alternatively, one could also refer to the number of veto players, i.e., the number of actors whose consent is needed to make a specific decision.

8 Additionally, the necessary majorities can be taken into account. As already shown by Buchanan and Tullock (1962) a high supermajority in one chamber can be equivalent to two simple majorities in two chambers.

9 Both Lintott (2009) and Straumann (2016) point out that many features that were taken for granted regarding the institution of dictatorship might not be historically accurate. Straumann, in particular, takes pains to uncover the historical evolution of the institution between its inception in 494BC and its abolition in 44BC.

10 It is true that some legislative measures are passed with a sunset clause, or are not renewed.

11 The data is from the Comparative Constitutions Project (see Elkins et al. 2009). The exact wording of the relevant variable is: ‘Does the constitution have provisions for calling a state of emergency?’ Countries with unwritten constitutions, such as the United Kingdom or New Zealand, are not taken into consideration here.

12 A reviewer pointed out that this claim may be premature as the ease with which states of emergency can be called would not depend on the number of conditions contained in the constitution but, rather, their comprehensiveness. Thus, a single very comprehensive clause may cover more events than more numerous, but narrowly limited clauses. Fair point. Within the realm of the Comparative Constitutional Project (Elkins et al. 2009), six events that can justify the declaration of a state of emergency have been coded. When comparing constitutional contents of 1950 with 2011, in five of the six dimensions significantly higher proportions were found in 2011. E.g. ‘war and aggression’ was explicitly mentioned in 32 percent of all emergency constitutions in 1950. Until 2011, the proportion had risen to 49 percent. I interpret this as evidence that the declaration of a state of emergency has, indeed, become easier.

13 Dragu and Polborn (2014) develop a game theoretic model focusing on this issue. There is solid evidence supporting this view (*see*, *e.g.*, Walsh and Piazza 2010).

14 A very detailed description complemented by an in-depth evaluation of both the strengths and the weaknesses of Locke’s approach can be found in Fatovic (2009, pp. 38–82).

15 Poole (2009) is yet another 21st-century perspective on Locke asking about the role of the prerogative in our age of statutes. He observes that the movement from prerogative to statute in crisis lawmaking is undertheorized and laments that formerly royal authority now exists ‘in a murky gray area between the authority of the legislature and the power of the executive’ (ibid., p. 254).

16 With regard to the current pandemic, there are voices claiming that emergency constitutions are not misused but, rather, underused (Pozen and Scheppele 2020), echoing Rousseau’s claim that the office of dictator was underused, rather than misused in Rome (IV, 6, 8 and 9).

17 The economic rationale underlying rights if spelled out in much more detail in Mueller (1991). Drawing on decision-making costs as introduced by Buchanan and Tullock (1962, Chapter 6), he shows that there could never be a need to define constitutional rights if all decision-making costs were zero. But, of course, they never are.

18 Legislation as a reaction to coronavirus was also very quick. In most non-Asian countries, the virus began to spread in February 2020. Here are some examples for when legislatures passed relevant laws: Canada: March 25; France: March 23; Ireland: March 20; Italy: February 23 (a decree law); Poland: March 2, and Ukraine: March 17 (all information from https://www.lexology.com/library/detail.aspx?g=d75c6657-a3f7-4312-b341-7ba8da835fd8; assessed on March 17, 2021).

19 ‘The precise details of an emergency cannot be anticipated, nor can one spell out what may take place in such a case, especially when it is truly a matter of an extreme emergency and of how it is to be eliminated. The precondition as well as the content of jurisdictional competence in such a case must necessarily be unlimited. From the liberal constitutional point of view, there would be no jurisdictional competence at all. The most guidance the constitution can provide is to indicate who can act in such a case’.

20 Over the last couple of decades, we have seen enormous technological changes that have substantially affected our lives, including personal computers and the world wide web. Many pieces of legislation have been passed to deal with new issues arising from the use of these technologies. But I am not aware of an argument that would demand constitutional change as a consequence of these developments. The question hence is: Are the developments supposedly threatening the life of a nation of such a different quality to justify constitutional changes?

21 The position that basic rights are luxury goods is also shared by Rossiter, whose study remains the most influential on constitutional dictatorship. He writes: ‘Civil liberties, free enterprise, constitutionalism, government by debate and compromise—these are strictly luxury products …’(2009, p. 5).

22 A reviewer pointed out that according to a number of international treaties, not all rights are derogable. This is, of course, correct. But it needs to be asked how such international treaties affect the behavior of nation-state governments. Bjørnskov and Voigt (2018b, p. 10) find that having ratified the International Covenant on Civil and Political Rights (ICCPR) does not affect the likelihood to declare a state of emergency. The Convention may, hence, perceived as not constraining government behavior. Countries that have ratified the Convention and declare a state of emergency are obliged to notify the UN. If the UN is not even notified, this can be interpreted as another indication of the limited relevance of the non-derogable core of the basic rights. According to the Centre for Civil and Political Rights, 87 governments had declared a state of emergency due to Covid 19. Of these, only 22 had notified the UN.

23 Korematsu v United States 323 U.S. 214 (1944).

24 Wu (2002) summarizes the findings of the report of the U.S. Commission on Wartime Relocation and Internment of Civilians (1983) by stating that ‘the internment had been motivated by wartime hysteria, racial prejudice, and failure of political leadership—not military necessity’.

25 There have been various attempts to evaluate the policy reactions of the U.S. government to 9/11 in terms of cost–benefit analyses. Mueller and Stewart (2011), for example, observe that the increase in expenditures on U.S. domestic homeland security over the decade since 9/11 is in excess of one trillion dollars, i.e., 100 billion dollars on an annual basis. Note that this number does not refer to total costs but only to increases in costs. Further note that it only refers to domestic expenditures and does not cover the costs of the interventions in Afghanistan and Iraq. The authors find that for these expenditures to be cost effective, they would have to deter 1,667 otherwise successful Times Square-type attacks per year or, equivalently, four such attacks every single day of the year! Blalock et al. (2009) deal with the unintended consequences of tougher regulation. Air travelers who do not want to lose time at the airport navagating tough controls might switch to the car for short-distance travel. It is estimated that this substitution behavior caused 327 additional driving deaths per month in late 2001. Although the effect weakened over time, the authors estimate that as many as 2,300 driving deaths may be attributable to the attacks.

26 It is possible to reframe the argument in a more continuous fashion. The economic justification of basic human rights refers to the protection of minorities. If there were completely homogenuous societies without any kind of minority, this sort of justification would not have bite. One could, hence, reframe the argument such that the justification of emergency constitutions becomes more difficult the more heterogenous the respective society.

27 The term ‘action bias’ was coined by Patt and Zeckhauser 2000 (Anthony Patt and Richard Zeckhauser, ‘Action Bias and Environmental Decisions’, 21(1) *Journal of Risk and Uncertainty* 45–72) and first applied to environmental issues. In the meantime, it has been applied to other areas including terrorism (see Cass Sunstein and Richard Zeckhauser, ‘Overreaction to Fearsome Risks’, *Environmental Resource Economics* 435–449 (2011)).

28 As has been shown with regard to counter-terrorism policies in Sect. 5.3 above.

29 This is, of course, not a new argument. Regarding the Roman emergency constitution, Montesquieu (1989) noted that the dictatorship was a means by which the plebeians could be intimidated.
